# Dementia, stroke, age, use of medical devices and antipsychotic drugs may increase the risk of nosocomial infections among elderly patients hospitalized at Neurology Clinics

**DOI:** 10.1038/s41598-023-46102-2

**Published:** 2023-10-31

**Authors:** Leonardo Biscetti, Valentina Cameriere, Tommaso Rossi, Eleonora Potente, Deborah Sabbatini, Francesco Bollettini, Simona Castellani, Letizia Ferrara, Roberta Galeazzi, Fabrizia Lattanzio, Mirko Di Rosa, Elisa Foresi, Giuseppe Pelliccioni

**Affiliations:** 1 Unit of Neurology, IRCCS INRCA-National Institute of Health and Science on Ageing, Ancona, Italy; 2 Medical Direction, IRCCS INRCA-National Institute of Health and Science on Ageing, Ancona, Italy; 3 Clinic of Laboratory and Precision Medicine, IRCCS INRCA-National Institute of Health and Science on Ageing, Ancona, Italy; 4 Scientific Direction, IRCCS INRCA-National Institute of Health and Science on Ageing, Ancona, Italy; 5 Centre for Biostatistics and Applied Geriatric Clinical Epidemiology, IRCCS INRCA-National Institute of Health and Science on Ageing, Ancona, Italy

**Keywords:** Neuroscience, Dementia

## Abstract

Healthcare-acquired infections (HCAI) represent a major health problem worldwide. Stroke and dementia are considered risk factors for HCAI. Preliminary data suggest that use of antipsychotic drugs also increase the risk for HCAI. Here, we performed a retrospective study aimed at investigating the major risk and protective factors for HCAI in a cohort of elderly subjects hospitalized at an Italian tertiary Neurology Clinics. We included all patients with age ≥ 65 years hospitalized at Neurology Clinics of National Institute on Ageing, Ancona, Italy from 1st January 2018 to 31st December 2021. For each patient, the following data were collected: age, sex, use of medical devices, comorbidities, use of antipsychotic medications, development of HCAI. We included 1543 patients (41.4% males; median age 85 years [80–89]). According to multivariable analysis, age, stroke, duration of urinary catheter placement (for all *p* < 0.001) and midline placement (*p* = 0.035) resulted to be risk factors for HCAI, Diabetes resulted to be a protective factor for pneumonia (*p* = 0.041), while dementia and nasogastric tube were risks factor for this condition (p = 0.022 and *p* < 0.001, respectively). Urinary catheter was a risk factor for urinary tract infections (*p* < 0.001). Duration of placement of vascular catheters and use of antipsychotic drugs resulted to significantly increase the risk for bloodstream infections. Stroke, age and use of medical devices were confirmed to be risk factors for HCAI. Antipsychotic drugs resulted to increase risk for bloodstream infections. Further prospective studies will be needed to confirm these findings.

## Introduction

Nosocomial infections, also known as healthcare-acquired infections (HCAI), represent one of the most important challenges which clinicians have to face in routine clinical practice. They cause a relevant burden for healthcare system worldwide, especially in developing countries^1^. HCAI often induce sepsis, a life-threatening syndrome caused by an abnormal host response to microbiological agents, with a mortality higher than 10%^2^. Indeed, HCAI often have a poorer outcome compared to community-acquired infections due to the combination of two factors: the frailer health status of hospitalized patients compared to non-hospitalized subjects and the higher prevalence of multidrug resistant bacteria in hospital setting in comparison with community setting.

An epidemiological study estimated 48.1 million cases of sepsis and 11 million sepsis-related deaths worldwide in 2017^3^; a meta-analysis based on studies conducted in high income countries and published in 2016 furnished an estimate of 30 millions of sepsis cases and 5.3 millions of deaths induced by sepsis every year worldwide^4^. These impressive numbers led us to understand the need of an accurate assessment of risk factors for nosocomial infections in order to implement effective preventive strategies.

Among HCAI, urinary tract infections and pneumonia are the most frequent^5^. Another kind of infection frequently detected in hospital setting and at high risk of complications is represented by bloodstream infections^6^.

A well-known risk factor for HCAI is the use of some medical devices, including urinary catheter and vascular catheters, especially for urinary tract infections and bloodstream infections^7–9^.

Nasogastric tube, a device frequently used in clinical practice to feed patients with dysphagia, may increase the risk of pneumonia^10^.

Additionally, some comorbidities, including diabetes mellitus (DM), may represent relevant risk factors for nosocomial infections^11^. Specifically, DM seems to significantly raise the risk of urinary infections^12^, while the implication of this condition as a risk factor for healthcare-acquired pneumonia is still controversial^13^.

With particular regard to Neurology Units, some medical conditions, including ischemic stroke and dementia, were also increasingly recognized as risk factors for nosocomial infections^14,15^*,* even if the underlying pathophysiological mechanisms linking neurological diseases and increased susceptibility to infections still need to be clarified in depth.

Furthermore, some studies suggest that antipsychotic drugs, frequently used in Neurology setting, might be an underestimated risk factor for infections, especially for pneumonia, but evidence in this field is still scarce^16^.

From a general point of view, one of the most recognized risk factors for poor outcome in case of nosocomial infection is advanced age^17^. However, the identification of the exact burden of each risk factor for HCAI among geriatric patients affected by neurological diseases is not available so far.

On this background, we performed a large retrospective study aimed at individuating the main risk and protective factors for nosocomial infections and their specific magnitude among elderly subjects hospitalized at a tertiary Neurology Clinics specialized in the management of geriatric patients. The general objective of this investigation is to give a contribution to the clinicians’ knowledge about this important—and still controversial—clinical issue, in order to improve the management of elderly patients affected by neurological diseases.

## Methods

### Cohort selection

This is a retrospective monocentric study based on the clinical data warehouse from the Italian National Institute of Health and Science on Ageing (Istituto Nazionale di Ricovero e Cura dell’Anziano a carattere scientifico, INRCA-IRCCS, Ancona, Italy). Here we included all hospitalized patients aged 65 years or over at the Neurology Clinic of INRCA-IRCCS—from 1st January 2018 to 31st December 2021. Before performing statistical analysis, all data were pseudo-anonymized in order to guarantee the compliance of the present study with the Italian law on privacy. The data extraction and management were run using Structured Query Language (SQL).

For each hospitalization, we collected all the following data: age, sex, date of admission, duration of hospitalization, duration of use of medical devices (central and peripheral vascular catheter, urinary catheter, nasogastric tube), use of antipsychotic drugs and all the diagnoses (from principal to sixth diagnosis) coded using the 9th revision of the International Classification of Diseases-Clinical Modification (ICD9-CM). The vascular catheters used in our setting were: peripheral intravenous catheter (PVC), central intravenous catheter (CVC), midline, power glide, peripherally inserted central catheter (PICC). Starting from the electronic database we analyzed the presence of comorbidities including diabetes mellitus and/or ischemic stroke and/or dementia, and development during hospitalization of healthcare-acquired pneumonia and/or nosocomial urinary tract infection and/or bloodstream infections. Dementia, now alternatively called neurocognitive major disorder, was diagnosed in all cases according to the Diagnostic and Statistical Manual of Mental Disorders fifth edition (DSM 5, 2013). Ischemic stroke was always diagnosed considering both clinical and neuroradiological data taken from brain computerized tomography (CT) or brain magnetic resonance imaging. In the statistical analysis, all types of dementia were grouped together (Alzheimer dementia, vascular dementia, frontotemporal dementia, dementia with Lewy bodies, Parkinson disease with dementia and other rare forms of major neurocognitive disorders), while patients with mild cognitive impairment were considered together with cognitively healthy subjects. Finally, clinicians working at the Neurology Unit of IRCCS INRCA (TR, VC, EP, FB, SC and DS) checked all medical records, in order to verify the correctness of obtained data by means of electronic warehouse and to insert missing information in dataset before performing statistical analysis.

### Diagnosis of infection

Diagnosis of pneumonia was made on the basis of both clinical and radiological features (chest radiography and/or chest CT), while urinary infections were diagnosed considering both clinical and microbiological data (i.e., clinical symptoms, including delirium, and positivity of urine culture according to cut-off used in our laboratory). Finally, bloodstream infections were diagnosed bearing in mind both clinical data (for instance, fever, hypotension and/or other signs or symptoms, including the increase of procalcitonin and/or c-reactive protein) and positivity of blood cultures in two samples for a single bacterium (necessarily, the same bacterium in both cultures). We considered as nosocomial infections only symptomatic infections treated with antibiotic or antimycotics drugs which started at least 48 h after admission.

### Statistical analysis

The normality in distribution of continuous variables was assessed via Shapiro–Wilk test and they were reported as either mean and standard deviation (SD), or median and interquartile range (IQR) as appropriate. Variables comparison between groups was performed, according to their distribution, by unpaired Student’s t test or Mann–Whitney U test. Categorical variables were expressed as absolute frequency and percentage and comparison between groups was performed via chi-square test.

In order to control for potential bias and confounding effects, three multivariable regression models were estimated for each outcome variable. To mitigate the potential risk of obtaining biased estimations due to rare events in our data, Firth penalized logistic regression models for pneumonia, urinary infection, bloodstream infections and at least one of the previous three kinds of infection were estimated. In Model 1, association of age, gender and main co-morbidities with study outcomes were reported; in Model 2, estimates of use of medical devices and antipsychotic administration adjusted for variables of Model 1 were reported. In the third model, length of medical devices’ usage and antipsychotic administration adjusted for variables of Model 1 were employed. With regard to medical devices, we considered nasogastric tube, urinary catheter and vascular catheters as potential risk factors for pneumonia, urinary tract infections and bloodstream infections, respectively. Odds Ratios (OR) for logistic models and 95% Confidence Interval (95%CI) for all estimates were reported.

Finally, in order to verify the robustness of obtained results, we performed a sensitivity analysis using the second and third model of multivariable analysis, in two subgroups of patients: subject with dementia vs subjects without dementia.

A 2-tailed *p* value of < 0.05 was considered significant. Data were analyzed using STATA version 15.1 (StataCorp, College Station, TX, USA).

### Ethical approval

The study was performed in accordance with the ethical standards laid down in the 1964 Declaration of Helsinki and its later amendments. Given the retrospective design of the study, patient consent collection and ethics committee approval were not applicable according to the policy of the Ethics Committee of IRCCS INRCA. All patients’ data were anonymized before statistical analysis.

## Results

### Demographic and clinical features of study cohort

In this study, 1543 patients with age of 65 years old or over were enrolled. Of those, 638 (41.4%) were male; the median age of the entire cohort was 85 years (80–89); the median duration of hospitalization was 11 days (7–17). Patients with dementia were 597 (38.8%), while subjects admitted for stroke were 313 (20.3%). Three hundred fifty patients (22.7%) in this study cohort were affected by diabetes mellitus; 228 subjects (14.8%) were treated with antipsychotic drugs during hospitalization due to severe behavioral disturbances not controlled by non-pharmacological approach.

CVC was applied to 22 patients (1.4%). Power glide was used only in 6 cases, while PVC was applied to 864 patients (56%). PICC was used in 33 cases (2.1%), while midline was applied to 44 subjects (2.9%). One-hundred sixty-four patients (10.6%) underwent urinary catheter placement and 57 patients (3.7%) were fed with nasogastric tube during hospitalization.

In the cohort, 159 patients (10.3%) were affected by healthcare-acquired pneumonia, while 155 (9.7%) developed nosocomial urinary tract infections and 17 (1.1%) bloodstream infections (some of them developed more than one infection during a single hospitalization).

In Table 1, all demographic and clinical data, including the duration of use of each medical device, were reported.

**Table 1:** Demographical and clinical features of the study cohort.

	Total	No Infection	Infection	*p*	No Pneumonia	Pneumonia	*p*
	n = 1,543	n = 1,263	n = 280		n = 1,384	n = 159	
Sex M, n(%)	639(41.4%)	523(41.4%)	116(41.4%)	0.995	556(40.2%)	83(52.2%)	**0.004**
Age, median(IQR)	85(80–89)	84(80–89)	87(83–91)	** < 0.001**	85(80–89)	87(83–90)	** < 0.001**
Dementia, n(%)	598(38.8%)	474(37.5%)	124(44.3%)	**0.036**	522(37.7%)	76(47.8%)	**0.013**
Stroke, n(%)	313(20.3%)	228(18.1%)	85(30.4%)	** < 0.001**	263(19%)	50(31.4%)	** < 0.001**
Diabetes, n(%)	350(22.7%)	294(23.3%)	56(20.0%)	0.236	324(23.4%)	26(16.4%)	**0.044**
Antipsychotic drugs, n(%)	228(14.8%)	176(13.9%)	52(18.6%)	**0.048**	200(14.5%)	28(17.6%)	0.288
Days of hospitalization, median(IQR)	11(7–17)	10(6–15)	19(11–27)	** < 0.001**	10(7–16)	21(12–32)	** < 0.001**
Devices							
CVC, n(%)	22(1.4%)	12(1.0%)	10(3.6%)	**0.001**	14(1%)	8(5%)	** < 0.001**
Days CVC, median(IQR)	3.5(2–9)	3.5(2–9)	4(3–7)	0.484	3(2–9)	6(3–14)	0.285
Power Glide, n(%)	6(0.4%)	5(0.4%)	1(0.4%)	0.925	5(0.4%)	1(0.6%)	0.608
Days Power Glide, median(IQR)	1(1–7)	1(1–7)	1(1–1)	0.480	1(1–7)	1(1–1)	0.480
PVC, n(%)	864(56%)	659(52.2%)	205(73.2%)	** < 0.001**	749(54.1%)	115(72.3%)	** < 0.001**
Days PVC, median(IQR)	5(3–9)	4(3–8)	6(3–13)	** < 0.001**	4(3–8)	6(3–15)	** < 0.001**
PICC, n(%)	33(2.1%)	22(1.7%)	11(3.9%)	**0.022**	22(1.6%)	11(6.9%)	** < 0.001**
Days PICC, median(IQR)	1(1–4)	1(1–10)	1(1–1)	0.343	1(1–10)	1(1–1)	0.343
Nasogastric tube, n(%)	57(3.7%)	26(2.1%)	31(11.1%)	** < 0.001**	34(2.5%)	23(14.5%)	** < 0.001**
Days nasogastric tube, median(IQR)	3(1–27)	2.5(1–13)	6(1–29)	0.771	1.5(1–10)	10(1–31)	0.144
Midline, n(%)	44(2.9%)	21(1.7%)	23(8.2%)	** < 0.001**	24(1.7%)	20(12.6%)	** < 0.001**
Days Midline, median(IQR)	2.5(1–7.5)	2(1–7)	4(1–11)	0.363	2.5(1–7)	3(1–8)	0.810
Urinary catheter, n(%)	164(10.6%)	77(6.1%)	87(31.1%)	** < 0.001**	109(7.9%)	55(34.6%)	** < 0.001**
Days Urinary catheter, median(IQR)	22.5(7–30)	14(4–29)	29(13–31)	**0.010**	15(7–29)	29(16–31)	**0.004**

	Total	No Urinary Infection	Urinary Infection	*p*	No Blood Infection	Blood Infection	*p*
	n = 1,543	n = 1,388	n = 155		n = 1,526	n = 17	
Sex M, n(%)	639(41.4%)	593(42.7%)	46(29.7%)	**0.002**	634(41.5%)	5(29.4%)	0.312
Age, median(IQR)	85(80–89)	84(80–89)	87(83–91)	** < 0.001**	85(80–89)	88(85–91)	0.075
Dementia, n(%)	598(38.8%)	538(38.8%)	60(38.7%)	0.990	592(38.8%)	6(35.3%)	0.768
Stroke, n(%)	313(20.3%)	267(19.2%)	46(29.7%)	**0.002**	307(20.1%)	6(35.3%)	0.122
Diabetes, n(%)	350(22.7%)	319(23.0%)	31(20.0%)	0.400	348(22.8%)	2(11.8%)	0.280
Antipsychotic drugs, n(%)	228(14.8%)	198(14.3%)	30(19.4%)	0.090	222(14.5%)	6(35.3%)	**0.017**
Days of hospitalization, median(IQR)	11(7–17)	10(7–16)	20(11–26)	** < 0.001**	11(7–17)	43(25–54)	** < 0.001**
Devices							
CVC, n(%)	22(1.4%)	18(1.3%)	4(2.6%)	0.201	20(1.3%)	2(11.8%)	** < 0.001**
Days CVC, median(IQR)	3.5(2–9)	3.5(2–9)	5(3–19)	0.465	3.5(2–8)	17(3–31)	0.419
Power Glide, n(%)	6(0.4%)	6(0.4%)	0(0%)	0.412	6(0.4%)	0(0%)	0.796
Days Power Glide, median(IQR)	1(1–7)	1(1–7)	–	–	1(1–7)	–	–
PVC, n(%)	864(56%)	747(53.8%)	117(75.5%)	** < 0.001**	852(55.8%)	12(70.6%)	0.223
Days PVC, median(IQR)	5(3–9)	4(3–9)	6(3–12)	**0.010**	4.5(3–9)	11(4–28.5)	**0.015**
PICC, n(%)	33(2.1%)	31(2.2%)	2(1.3%)	0.441	33(2.2%)	0(0%)	0.540
Days PICC, median(IQR)	1(1–4)	1(1–4)	7.5(1–14)	0.532	1(1–4)	–	–
Nasogastric tube, n(%)	57(3.7%)	40(2.9%)	17(11%)	** < 0.001**	50(3.3%)	7(41.2%)	** < 0.001**
Days SNG, median(IQR)	3(1–27)	2(1–14.5)	10(1–34)	0.157	2.5(1–16)	29(1–35)	**0.015**
Midline, n(%)	44(2.9%)	33(2.4%)	11(7.1%)	**0.001**	41(2.7%)	3(17.6%)	** < 0.001**
Days Midline, median(IQR)	2.5(1–7.5)	2(1–6)	8(2–17)	**0.021**	2(1–7)	20(2–29)	0.138
Urinary catheter, n(%)	164(10.6%)	110(7.9%)	54(34.8%)	** < 0.001**	153(10.0%)	11(64.7%)	** < 0.001**
Days Urinary catheter, median(IQR)	22.5(7–30)	21.5(7–30)	23(9–31)	0.498	21(7–30)	29(29–50)	0.064

*CVC* central intravenous catheter, *PVC* peripheral intravenous catheter, *PICC* peripherally inserted central catheter.

Significant values are in [bold].

### Univariate analysis: putative risk and protective factors for nosocomial infections

In the present analysis, urinary tract infections, bloodstream infections and pneumonia are grouped together as nosocomial infections. According to univariate analysis, dementia (*p* = 0.037), antipsychotic drugs (*p* = 0.043) age, stroke, duration of hospitalization, PVC use and duration of its placement, nasogastric tube and urinary catheter (for all of which, *p* < 0.001) resulted to be significant risk factors for nosocomial infections. Also use of CVC (*p* = 0.001), PICC (*p* = 0.021) and duration of urinary catheter placement (*p* = 0.010)were found to be associated to higher risk of HCAI.

### Univariate analysis: putative risk and protective factors for healthcare-acquired pneumonia

After univariate analysis, male sex (*p* = 0.004), dementia (*p* = 0.013), age, stroke, duration of hospitalization and nasogastric tube (for all of which, *p* < 0.001) resulted to be associated to an increased risk of healthcare-acquired pneumonia.

Interestingly, DM resulted instead a protective factor for this condition (*p* = 0.044).

### Univariate analysis: putative risk and protective factors for nosocomial urinary tract infections

After univariate analysis, stroke (*p* = 0.002), age, duration of hospitalization and urinary catheter (for all of which, *p* < 0.001) resulted to be significantly associated to an increased risk of nosocomial urinary tract infections. On the other hand, male sex resulted to be a protective risk factor for this disease (*p* = 0.002).

### Univariate analysis: putative risk and protective factors for nosocomial bloodstream infections

After univariate analysis, duration of hospitalization, use of CVC, midline use (for all of which, *p* < 0.001) and duration of PVC placement (*p* = 0.015) resulted to be significantly associated to an increased risk of bloodstream infections. Interestingly, also use of antipsychotic drugs was found as a risk factor for this kind of infection (*p* = 0.017).

### Multivariable analysis: risk and protective factors for nosocomial infections

In the first model of multivariable analysis, stroke (OR1.82, 95% CI 1.34–2.47, *p* < 0.001)

resulted to be the strongest risk factors for nosocomial infections; conversely, age resulted to increase only slightly the risk of HCAI (OR 1.07, 95% CI 1.04–1.09, *p* < 0.001).

In the second and third model of multivariable analysis, the burden of the above-mentioned risk factors resulted to be very similar with respect to first model. In the second model, also PVC (OR 1.66, 95% CI 1.21 − 2.27, *p* < 0.001), midline (OR 2.42, 95% CI 1.19–4.90, *p* = 0.014), nasogastric tube (OR 2.17, 95% CI 1.17–4.02, *p* = 0.014) and urinary catheter (OR 5.10, 95% CI 3.50–7.42, *p* < 0.001) resulted to be very strong risk factors for HCAI. Finally, in the third model taking into account the duration of medical device use, in addition to age and stroke also duration of urinary catheter and midline placement (OR 1.07, 95% CI 1.05–1.09, *p* < 0.001 and OR 1.13, 95% CI 1.01–1.26, *p* < 0.035, respectively) resulted to be risk factors for nosocomial infections.

### Multivariable analysis: risk and protective factors for healthcare-acquired pneumonia

In the first model of multivariable analysis, male sex (OR 1.99, 95% CI 1.42–2.81, *p* < 0.001), dementia (OR 1.58, 95% CI 1.12–2.22, *p = 0.009*) and stroke (OR 1.95, 95% CI 1.34–2.85, *p* = 0.001) resulted to be the strongest risk factors for healthcare-acquired pneumonia, whereas age resulted to be only a weak risk factor (OR 1.06, 95% CI 1.03–1.09, *p* < 0.001). Finally, diabetes (OR 0.62, 95% CI 0.39–0.96, *p* = 0.034) resulted to be a protective factor for this condition.

In the second model of multivariable analysis, taking into account the use vs not-use of medical devices, a strong trend supporting a protective role for diabetes nearly reaching statistical significance was observed (OR 0.64, 95% C.I. 0.41–1.00, *p* = 0.051). On the other hand, nasogastric tube resulted to be a very strong risk factor for nosocomial pneumonia (OR 5.87, 95% CI 3.26–10.54, *p* < 0.001).

Finally, in the third model, in addition to male sex, dementia, stroke and age, also the duration of nasogastric tube placement resulted to be weakly associated to an increased risk of healthcare-acquired pneumonia (OR 1.06, 95% CI 1.03–1.08, *p* < 0.001). Also, in this model diabetes was confirmed as a protective factor (OR 0.62, 95% C.I. 0.4–0.098, *p* = 0.041) (see Table 2).

**Table 2:** Risk and protective factors for nosocomial infections: all, pneumonia, urinary-tract infection and bloodstream infection.

Model 1			Model 2			Model 3		
	OR(95%CI)	*p*		OR(95%CI)	*p*		OR(95%CI)	*p*
Outcome: nosocomial infections								
Sex M	1.17(0.89–1.54)	0.252	Sex M	1.29(0.97–1.73)	0.082	Sex M	1.19(0.90–1.58)	0.231
Age	1.07(1.04–1.09)	** < 0.001**	Age	1.05(1.03–1.08)	** < 0.001**	Age	1.06(1.04–1.09)	** < 0.001**
Dementia	1.31(1.00–1.71)	0.054	Dementia	1.13(0.84–1.53)	0.413	Dementia	1.25(0.93–1.68)	0.133
Stroke	1.81(1.33–2.45)	** < 0.001**	Stroke	1.71(1.23–2.38)	**0.001**	Stroke	1.79(1.30–2.47)	** < 0.001**
Diabetes	0.84(0.61–1.17)	0.304	Diabetes	0.90(0.63–1.27)	0.538	Diabetes	0.90(0.64–1.27)	0.555
			Antipsychotic drugs	1.07(0.73–1.57)	0.726	Antipsychotic drugs	1.13(0.77–1.65)	0.543
			CVC	2.91(1.18–7.18)	**0.021**	days CVC	1.02(0.98–1.06)	0.437
			Power Glide	0.28(0.04–1.85)	0.188	days Power Glide	0.70(0.42–1.16)	0.162
			PVC	1.65(1.20–2.25)	**0.002**	days PVC	1.00(1.00–1.00)	0.690
			PICC	0.74(0.32–1.70)	0.474	days PICC	1.00(0.98–1.02)	0.909
			Nasogastric tube	2.35(1.27–4.32)	**0.006**	Days Nasogastric tube	1.00(0.97–1.03)	0.865
			Midline	2.39(1.18–4.84)	**0.016**	days Midline	1.13(1.01–1.26)	**0.036**
			Urinary Catheter	5.01(3.45–7.30)	** < 0.001**	Days Urinary Catheter	1.07(1.05–1.09)	** < 0.001**
_cons	0.00(0.00–0.00)	0.000	_cons	0.00(0.00–0.01)	< 0.001	_cons	0.00(0.00–0.00)	< 0.001
Outcome: pneumonia								
Sex M	1.99(1.42–2.81)	** < 0.001**	Sex M	2.19(1.54–3.11)	** < 0.001**	Sex M	2.02(1.43–2.85)	** < 0.001**
Age	1.06(1.03–1.09)	** < 0.001**	Age	1.06(1.03–1.08)	** < 0.001**	Age	1.06(1.03–1.09)	** < 0.001**
Dementia	1.58(1.12–2.22)	**0.009**	Dementia	1.52(1.06–2.18)	**0.022**	Dementia	1.55(1.09–2.21)	**0.016**
Stroke	1.95(1.34–2.85)	**0.001**	Stroke	1.79(1.22–2.64)	**0.003**	Stroke	1.90(1.30–2.78)	**0.001**
Diabetes	0.62(0.39–0.96)	**0.034**	Diabetes	0.64(0.41–1.00)	0.051	Diabetes	0.62(0.40–0.98)	**0.041**
			Antipsychotic drugs	0.96(0.60–1.53)	0.860	Antipsychotic drugs	0.95(0.60–1.51)	0.824
			Nasogastric tube	5.87(3.26–10.54)	** < 0.001**	days Nasogastric tube	1.06(1.03–1.08)	** < 0.001**
_cons	0.00(0.00–0.00)	< 0.001	_cons	0.00(0.00–0.00)	< 0.001	_cons	0.00(0.00–0.00)	< 0.001
Outcome: urinary–tract infection								
Sex M	0.63(0.44–0.91)	**0.014**	Sex M	0.65(0.45–0.95)	**0.027**	Sex M	0.63(0.44–0.92)	**0.016**
Age	1.06(1.03–1.09)	** < 0.001**	Age	1.05(1.02–1.08)	**0.001**	Age	1.06(1.03–1.09)	** < 0.001**
Dementia	0.93(0.65–1.31)	0.664	Dementia	0.83(0.57–1.21)	0.338	Dementia	0.87(0.60–1.26)	0.450
Stroke	1.52(1.03–2.22)	**0.033**	Stroke	1.52(1.03–2.26)	**0.037**	Stroke	1.47(0.99–2.18)	0.056
Diabetes	0.91(0.60–1.38)	0.651	Diabetes	0.94(0.61–1.46)	0.795	Diabetes	0.95(0.61–1.46)	0.805
			Antipsychotic drugs	1.33(0.84–2.12)	0.225	Antipsychotic drugs	1.34(0.85–2.12)	0.214
			Urinary Catheter	5.42(3.68–8.00)	** < 0.001**	Days Urinary Catheter	1.05(1.04–1.07)	** < 0.001**
_cons	0.00(0.00–0.01)	< 0.001	_cons	0.00(0.00–0.02)	< 0.001	_cons	0.00(0.00–0.02)	< 0.001
Outcome: bloodstream infection								
Sex M	0.70(0.25–1.95)	0.497	Sex M	0.74(0.27–2.08)	0.573	Sex M	0.75(0.26–2.14)	0.586
Age	1.05(0.97–1.13)	0.243	Age	1.05(0.97–1.13)	0.255	Age	1.03(0.95–1.12)	0.456
Dementia	0.84(0.32–2.26)	0.736	Dementia	0.55(0.19–1.60)	0.270	Dementia	0.54(0.18–1.65)	0.280
Stroke	1.99(0.73–5.43)	0.178	Stroke	1.92(0.70–5.26)	0.205	Stroke	2.04(0.72–5.73)	0.178
Diabetes	0.55(0.14–2.14)	0.393	Diabetes	0.66(0.17–2.54)	0.542	Diabetes	0.55(0.13–2.32)	0.419
			Antipsychotic drugs	3.45(1.20–9.95)	**0.022**	Antipsychotic drugs	3.50(1.15–10.66)	**0.028**
			CVC	9.47(2.22–40.28)	**0.002**	days CVC	1.07(1.02–1.12)	**0.003**
			Power Glide	6.20(0.29–131.61)	0.242	days Power Glide	1.70(1.06–2.72)	**0.029**
			PVC	1.22(0.43–3.48)	0.705	days PVC	1.00(1.00–1.00)	0.051
			PICC	0.73(0.04–13.18)	0.832	days PICC	1.02(1.00–1.03)	0.092
			Midline	6.75(1.95–23.37)	**0.003**	days Midline	1.22(1.09–1.36)	**0.001**
_cons	0.00(0.00–0.39)	0.025	_cons	0.00(0.00–0.29)	0.021	_cons	0.00(0.00–1.20)	0.056

*CVC* central intravenous catheter, *PVC* peripheral intravenous catheter, *PICC* peripherally inserted central catheter.

Significant values are in [bold].

### Multivariable analysis: risk and protective factors for nosocomial urinary tract infections

In the first model of multivariable analysis, male sex resulted to be a protective factor (OR 0.63, 95% CI 0.44–0.91, *p* = 0.014), whereas age and stroke were found as risk factors for nosocomial urinary tract infections (OR 1.06, 95% CI 1.03–1.09, *p* < 0.001 and OR 1.52, 95% CI 1.03–2.22, *p* = 0.033, respectively).

In the second model of multivariable analysis, the same risk and protective factors detected by the first model were found. As expected, in this model urinary catheter resulted to be the major risk factor for nosocomial urinary tract infections in our cohort (OR 5.42, 95% CI 3.68–8.00, *p* < 0.001).

Finally, in the third model, male sex and age were confirmed to be a protective and a risk factor for nosocomial urinary tract infections, respectively; furthermore, in this model, the duration of placement of urinary catheter resulted to be a risk factor for this condition (OR 1.05, 95% CI 1.03–1.07, *p* < 0.001) (see Table 2).

### Multivariable analysis: risk and protective factors for bloodstream infections

In the first model of multivariable analysis, no risk or protective factors for bloodstream infections were identified.

In the second model of multivariable analysis, antipsychotic drugs (OR 3.45, 95% CI 1.2–9.95, *p* = 0.022), CVC (OR 9.47, 95% CI 2.22–40.28, *p* = 0.002) and midline (OR 6.75, 95% CI 1.95–23.37, *p* = 0.003) resulted to be strong risk factors for bloodstream infections.

In the third model of multivariable analysis, antipsychotic drugs were confirmed to be associated to an increased risk of bloodstream infections. Furthermore, in this model, the duration of CVC (OR 1.07, 95% CI 1.02–1.12, *p* = 0.003), power glide (OR 1.70, 95% CI 1.06–2.72, *p* = 0.029) and midline (OR 1.22, 95% CI 1.09–1.36, *p* = 0.001) placement were found as weak risk factors for bloodstream infections.

In Table 2, all results taken from multivariable analysis were reported.

### Sensitivity analysis: risk factors and protective factors for total nosocomial infections, healthcare-acquired pneumonia, nosocomial urinary tract infections and bloodstream infections in patients with and without dementia

According to sensitivity analysis, stroke, age and urinary catheter were found to be associated to an increased risk for nosocomial infections in both patients with and without dementia. The duration of urinary catheter placement also was found to increase the risk for nosocomial infections in both groups..

Regarding healthcare-acquired pneumonia, male sex and duration of nasogastric placement were found to be associated to increased prevalence of this condition in both patients with and without dementia. Stroke significantly increased the risk of nosocomial pneumonia only in patients with dementia, while nasogastric tube resulted to be a significant risk factor for this condition only in patients without dementia.

With respect to nosocomial urinary tract infections, age and urinary catheter resulted to be a weak and a strong risk factor respectively in both patients with and without dementia. The duration of urinary catheter placement was also found to be associated to an increased rate of this kind of infection in both groups. Male sex resulted to be a significant protective factor only in patients with dementia, even if also in patients without dementia a similar trend was observed.

Finally, according to sensitivity analysis, only duration of midline placement was found to be a significant risk factor for bloodstream infections in both patients with and without dementia. Antipsychotic drugs, PVC and midline resulted to be associated with a statistically significant increase of prevalence of this condition only in patients without dementia.

Results taken from sensitivity analysis were summarized in Table 3.

**Table 3:** Risk factors and protective factors in patients with and without dementia.

Model 2					Model 3				
	No Dementia		Dementia			No Dementia		Dementia	
	OR(95%CI)	*p*	OR(95%CI)	*p*		OR(95%CI)	*p*	OR(95%CI)	*p*
Outcome: nosocomial infections									
Sex M	1.50(1.01–2.23)	**0.046**	1.25(0.81–1.94)	0.309	Sex M	1.33(0.90–1.95)	0.152	1.23(0.80–1.89)	0.350
Age	1.06(1.03–1.10)	** < 0.001**	1.04(1.00–1.07)	**0.048**	Age	1.08(1.05–1.11)	** < 0.001**	1.04(1.00–1.07)	**0.026**
Stroke	1.38(0.89–2.13)	0.146	2.48(1.47–4.19)	**0.001**	Stroke	1.51(0.99–2.29)	0.054	2.55(1.53–4.27)	** < 0.001**
Diabetes	0.90(0.56–1.43)	0.648	0.84(0.49–1.44)	0.524	Diabetes	0.88(0.56–1.39)	0.584	0.89(0.52–1.53)	0.683
Antipsychotic drugs	1.16(0.58–2.28)	0.677	1.03(0.65–1.63)	0.897	Antipsychotic drugs	1.28(0.66–2.48)	0.466	1.07(0.68–1.69)	0.765
CVC	3.01(0.82–11.09)	0.097	2.81(0.81–9.79)	0.104	days CVC	1.01(0.97–1.05)	0.680	1.06(0.95–1.18)	0.304
Power Glide	0.56(0.05–5.90)	0.630	0.18(0.01–3.71)	0.270	days Power Glide	1.06(0.05–20.76)	0.969	0.81(0.48–1.36)	0.420
PVC	1.69(1.12–2.55)	**0.013**	1.52(0.94–2.46)	0.090	days PVC	1.00(1.00–1.00)	0.693	1.00(0.99–1.01)	0.536
PICC	0.55(0.17–1.75)	0.309	1.00(0.31–3.26)	0.997	days PICC	1.00(0.98–1.02)	0.987	1.01(0.91–1.13)	0.796
Nasogastric tube	6.22(2.40–16.08)	** < 0.001**	0.99(0.41–2.42)	0.990	days Nasogastric tube	1.01(0.95–1.07)	0.846	1.01(0.97–1.04)	0.782
Midline	4.41(1.49–13.03)	**0.007**	1.85(0.74–4.58)	0.186	days Midline	1.36(1.08–1.70)	**0.008**	1.05(0.94–1.16)	0.376
Urinary catheter	7.17(4.21–12.22)	** < 0.001**	3.35(1.94–5.78)	** < 0.001**	days Urinary catheter	1.09(1.07–1.11)	** < 0.001**	1.04(1.02–1.06)	** < 0.001**
_cons	0.00(0.00–0.00)	< 0.001	0.01(0.00–0.11)	0.001	_cons	0.00(0.00–0.00)	< 0.001	0.01(0.00–0.11)	0.001
Outcome: pneumonia									
Sex M	2.02(1.23–3.32)	**0.005**	2.69(1.62–4.48)	** < 0.001**	Sex M	1.74(1.09–2.79)	**0.021**	2.61(1.57–4.34)	** < 0.001**
Age	1.08(1.04–1.12)	** < 0.001**	1.03(0.99–1.07)	0.166	Age	1.08(1.05–1.13)	** < 0.001**	1.03(0.99–1.07)	0.164
Stroke	1.27(0.75–2.15)	0.374	3.03(1.67–5.48)	** < 0.001**	Stroke	1.37(0.82–2.27)	0.227	3.16(1.75–5.72)	** < 0.001**
Diabetes	0.59(0.31–1.11)	0.101	0.66(0.34–1.29)	0.221	Diabetes	0.61(0.33–1.14)	0.121	0.66(0.34–1.28)	0.217
Antipsychotic drugs	1.13(0.50–2.57)	0.776	0.91(0.52–1.58)	0.731	Antipsychotic drugs	1.01(0.44–2.31)	0.985	0.90(0.52–1.58)	0.726
Nasogastric tube	16.06(7.05–36.57)	** < 0.001**	1.98(0.79–4.99)	0.146	days Nasogastric tube	1.09(1.04–1.15)	** < 0.001**	1.03(1.00–1.06)	**0.046**
_cons	0.00(0.00–0.00)	< 0.001	0.00(0.00–0.10)	0.001	_cons	0.00(0.00–0.00)	< 0.001	0.00(0.00–0.10)	0.001
Outcome: urinary tract infections									
Sex M	0.78(0.49–1.25)	0.311	0.53(0.28–1.00)	**0.049**	Sex M	0.75(0.47–1.19)	0.222	0.53(0.28–1.00)	**0.049**
Age	1.05(1.01–1.08)	**0.010**	1.05(1.00–1.10)	**0.035**	Age	1.06(1.02–1.09)	**0.002**	1.05(1.01–1.10)	**0.029**
Stroke	1.47(0.90–2.40)	0.123	1.64(0.84–3.19)	0.145	Stroke	1.33(0.81–2.18)	0.256	1.72(0.89–3.33)	0.104
Diabetes	0.92(0.53–1.58)	0.752	0.99(0.48–2.04)	0.972	Diabetes	0.92(0.53–1.58)	0.761	0.96(0.47–1.98)	0.922
Antipsychotic drugs	1.42(0.67–2.98)	0.358	1.30(0.73–2.33)	0.375	Antipsychotic drugs	1.48(0.72–3.06)	0.290	1.28(0.72–2.29)	0.397
Urinary catheter	7.10(4.28–11.78)	** < 0.001**	3.81(2.05–7.07)	** < 0.001**	Days Urinary catheter	1.07(1.05–1.09)	** < 0.001**	1.04(1.01–1.06)	**0.004**
_cons	0.00(0.00–0.05)	< 0.001	0.00(0.00–0.16)	0.005	_cons	0.00(0.00–0.03)	< 0.001	0.00(0.00–0.15)	0.005
Outcome: bloodstream infections									
Sex M	0.98(0.28–3.39)	0.977	0.50(0.08–3.03)	0.454	Sex M	0.93(0.27–3.27)	0.912	0.58(0.10–3.50)	0.554
Age	1.05(0.95–1.15)	0.361	1.06(0.92–1.22)	0.421	Age	1.01(0.92–1.10)	0.885	1.07(0.92–1.25)	0.376
Stroke	2.33(0.70–7.81)	0.170	1.12(0.17–7.23)	0.907	Stroke	2.59(0.77–8.69)	0.125	1.14(0.17–7.59)	0.894
Diabetes	0.54(0.09–3.09)	0.488	1.42(0.23–8.73)	0.703	Diabetes	0.34(0.05–2.36)	0.273	1.53(0.23–10.03)	0.655
Antipsychotic drugs	5.06(1.36–18.77)	0.015	2.24(0.51–9.78)	0.284	Antipsychotic drugs	5.72(1.54–21.26)	**0.009**	1.90(0.39–9.26)	0.429
CVC	13.11(2.54–67.76)	**0.002**	3.97(0.19–84.93)	0.378	days CVC	1.08(1.03–1.14)	**0.002**	1.16(0.98–1.36)	0.080
Power Glide	5.85(0.18–190.96)	0.320	7.65(0.30–192.31)	0.216	days Power Glide	6.96(0.21–225.96)	0.275	1.40(0.85–2.30)	0.188
PVC	1.35(0.36–5.09)	0.662	0.77(0.16–3.60)	0.736	days PVC	1.00(1.00–1.00)	**0.035**	1.01(1.00–1.02)	0.173
PICC	0.99(0.05–18.66)	0.993	2.26(0.10–50.18)	0.605	days PICC	1.01(1.00–1.03)	0.142	1.13(0.99–1.28)	0.073
Midline	9.05(1.77–46.21)	**0.008**	5.42(0.84–34.88)	0.075	days Midline	1.27(1.08–1.48)	**0.004**	1.18(1.05–1.32)	**0.007**
_cons	0.00(0.00–0.85)	0.046	0.00(0.00–64.60)	0.187	_cons	0.01(0.00–15.65)	0.198	0.00(0.00–47.92)	0.157

*CVC* central intravenous catheter, *PVC* peripheral intravenous catheter, *PICC* peripherally inserted central catheter.

Significant values are in [bold].

Finally, in supplementary materials, we have included multivariable models without overlapping diagnoses in order to analyze the specific impact of each disease net of the influence of comorbidities. In these models, the results are quite similar to those obtained in models including all patients (with and without comorbidities), with two exceptions: 1) dementia lost its role as risk factor for infections; 2) diabetes did not result a protective factor for healthcare- acquired pneumonia.

## Discussion

This retrospective study is aimed at individuating the main risk and protective factors for nosocomial infections in a relatively large cohort of elderly patients hospitalized at a Tertiary Neurology Clinic in Italy. The prevalence of HCAI was significantly higher in our cohort than that reported in previously published epidemiological studies performed in different geographic areas. For instance, in a large study made in the Netherlands in 2007–2008, the global prevalence of HCAI was 7%^18^, in two previous studies performed in Italy it resulted 4.9% and 7%, respectively^19,20^, and in a recent survey based on global data from European Union was 6.5%^21^, while in our cohort the number of nosocomial infections/number of hospitalizations ratio was 18%. This difference may be explained by the different typology of subjects included in our investigation with respect to patients included in the above-mentioned studies, which were focused on heterogenous hospital settings and not only on a geriatric neurological setting. If this interpretation is correct, the discrepancy between our data and data taken from the literature might suggest that the prevalence of nosocomial infections among elderly patients is tendentially higher than among young subjects, as previously reported^22,23^, and that elderly patients with neurological diseases could be particularly susceptible to HCAI. A further possible explanation of this discrepancy is the major duration of hospitalization in our cohort (median 11 days, 7–17), again related to the specific category of patients included in the present study (geriatric patients often with disabling neurological diseases), compared to the average length of stay in hospital in Italy and in other European countries^24^. The duration of hospitalization and the prevalence of nosocomial infections are indeed synergistically interrelated, since higher length of stay in hospital is obviously associated to increased risk of nosocomial infections and nosocomial infections in turn generally increase the duration of hospitalization.

Our investigation confirmed the role of medical devices as important risk factors for HCAI among elderly people. Specifically, as expected, both multivariable and sensitivity analysis showed that nasogastric tube, urinary catheter and vascular catheters were very strong risk factors for healthcare-acquired pneumonia, urinary infections and bloodstream infections, respectively. These results are not surprising and in strict accordance with the scientific literature on this topic^25–28^.

Among the elderly patients included in our cohort, older age resulted to be associated with a slight, but statistically significant, higher rate of nosocomial infections globally considered, healthcare-acquired pneumonia and urinary infections. This result was found by both univariate and different models of multivariable analysis and confirmed by sensitivity analysis in both people with and without dementia, thus appearing sufficiently robust,.

In our study, we did not find a statistically significant association between dementia and the risk of nosocomial infections, but only a trend in this regard (however, in the first model of multivariable analysis, the effect of dementia on the the risk of HCAI nearly reached the statistical significance with *p* = 0.055). More in detail, our study showed a clear association between dementia and pneumonia, while no other relationship was found between dementia and urinary tract-infections. The link between dementia and a higher rate of healthcare-acquired pneumonia found in our study is largely supported by the literature^29^. This association was probably driven by an increased rate of aspiration pneumonia among patients with dementia, compared to subjects without. This difference, in turn, may be probably due to a higher rate of dysphagia in subjects with major neurocognitive disorders, compared to cognitively healthy subjects and patients with mild cognitive impairment. However, we are not able to verify the correctness of this interpretation, due to the lack of data needed to make a precise classification of pneumonia cases registered in our study.

With respect to the possible relationship between dementia and nosocomial urinary treat infections in geriatric setting, it is still a quite controversial matter in scientific literature. In fact, to our knowledge, on one hand, the majority of studies supported a link between these two conditions^30,31^, but, on the other hand, a large investigation specifically focused on geriatric patients denied this association, even reporting a paradoxical protective effect of dementia in this regard, probably due to a greater difficulty of patients with major neurocognitive disorder in explaining their urinary disturbances, compared to cognitively healthy older subjects^32^. In our study, we did not find any association between dementia and nosocomial urinary tract infections. To this regard, it is important to underline that many nondemented patients hospitalized at Neurology Clinics, compared with subjects without neurological diseases, may be at higher risk of urinary tract infections, due to several predisposing conditions, such as neurogenic bladder^33^. Therefore, it is possible to hypothesize that the absence of any significant effect of dementia on nosocomial urinary tract infections in our study might be caused, at least in part, by a particularly high prevalence of this kind of infection in neurological patients without dementia (about 10%), compared to the prevalence reported in other studies not specifically focused on neurological settings (about 2%)^34^.

The present research supported a strong effect of stroke on the risk of nosocomial infections (globally considered), healthcare-acquired pneumonia and nosocomial urinary tract infections among geriatric patients with neurological disorders. Indeed, according to multivariable analysis, stroke almost doubled the risk of HCAI in our cohort. Specifically, about 30% of patients with stroke involved in the present study developed at least one nosocomial infection during hospitalization, about 15% were affected by nosocomial urinary tract infections, and 15% by healthcare-acquired pneumonia. These data are in good agreement with a meta-analysis published in 2011 and focused on the prevalence of infections after stroke^14^. Many mechanisms could explain the link between stroke and infections. First of all, many patients with stroke developed dysphagia, which in turn is a strong risk factor for pneumonia^35^. Secondly, bladder dysfunction (i.e., urinary incontinence and retention), a well-known risk factor for urinary tract infections, is very frequent after stroke, occurring in 29 to 58% of patients^36^. Finally, a growing body of evidence from both pre-clinical and clinical studies supported the occurrence of a significant systemic immunodepression after stroke, with a consequent increase of susceptibility to infections^37^.

According to sensitivity analysis, the impact of stroke on the risk of infection was relevant in both people with and without dementia, but among people affected by major neurocognitive disorders it seems to be of a greater extent, mainly with respect to the risk of nosocomial pneumonia. This finding suggests that dementia and stroke produce a synergistic effect on the risk for this kind of infection, probably due to a higher neurological deterioration after stroke in demented patients, compared to non-demented subjects, as suggested by a recent multicenter study^38^. This in turn may explain a higher rate of dysphagia in stroke patients with dementia compared to stroke patients without dementia and, consequently, a different rate of nosocomial pneumonia in the two groups. The synergistic effect between stroke and dementia is also indirectly supported by models including patients without overlapping diagnoses. These models indeed did not identify dementia as statistically significant risk factor for healthcare acquired pneumonia and for nosocomial infections (globally considered) in patients without comorbidities, probably because in our setting dementia per se does not relevantly increase this risk in subjects without overlapping diagnoses, but acts mostly as adding risk factor in patients with other predisposing medical conditions (first of all, stroke).

Our study has also shown a significant impact of sex in determining the risk of nosocomial urinary tract infections and pneumonia. More in details, according to multivariable analysis, male sex resulted to be a protective factor for urinary tract infections and a risk factor for nosocomial pneumonia. These data are in agreement with previous studies. Indeed, hospital-acquired pneumonia seems to be more common in men^39^ and male sex has been reported to be a risk factor for aspiration pneumonia in older patients^40^. Moreover, urinary tract infections are more frequent in women than in men^34^.

From a general point of view, we did not find any significant effect of male sex with respect to the risk of nosocomial infections (globally considered). This finding is not coherent with the scientific literature, according to which males are more susceptible to bacterial infections (globally considered)^41^. In our opinion, this discrepancy could be explained taking into account two factors: i) in our study, we did not investigate all possible kinds of bacterial infections, but only pneumonia, urinary tract infections and bloodstream infections (for instance, we did not include in final analysis gastrointestinal infections, which are typically more frequent in men^42^; ii) in our setting, urinary tract infections, which are typically more frequent in women, resulted to be as frequent as pneumonia, while nosocomial pneumonia cases (which are prevalent in men) are generally reported to be more frequent than nosocomial urinary tract infections^43^. For these reasons, the absence of any effect of male sex on the risk of nosocomial infections found in our study appears not to be generalizable.

According to sensitivity analysis, male sex resulted to be a significant risk factor for pneumonia in both patients with and without dementia and a protective factor for urinary infections only in patients with dementia. This latter finding is quite surprising and needs to be briefly analyzed. To this regard, we cannot exclude that the different impact of male sex on the risk of urinary tract infections among patients with and without dementia found in our study could be caused by the presence of confounding factors, for instance a casual different incidence of benign prostatic hypertrophy (BPH) in the two groups. To this regard, it is very difficult to measure the potential impact of this confounding factor, because BPH prevalence is greatly underestimated in general population^44^. Furthermore, it is important to underline that in our study male subjects without dementia with urinary dysfunction due to neurogenic bladder and therefore at high risk of urinary tract infections, are probably overrepresented compared to the general older population, thus potentially masking the gender effect on this kind of infection. Considering these factors, our results from sensitivity analysis about the impact of male sex on urinary tract infections should be interpreted with great caution, mainly in the view of their generalization.

In the present study, we also investigated the impact of diabetes on healthcare-acquired infections. According to multivariable analysis, we did not find any effect of diabetes on the risk of nosocomial urinary tract infections. This result was already reported by another study^45^, but it is not in agreement with the majority of papers on this topic, as summarized by a recent meta-analysis^46^. Again, this discrepancy may be explained considering the specific setting of the present study: a tertiary Neurology Clinic specialized in managing geriatric patients. In this context, different confounding factors, including a particularly high representation of patients with neurogenic bladder due to several conditions other than diabetes (for instance parkinsonian syndromes and myelopathy), may probably mask the effect of diabetes on the risk of this kind of infection.

The present investigation also showed a protective effect of diabetes with respect to the risk of nosocomial pneumonia. This result proved to be statistically significant according to different models of multivariable analysis, but not according to sensitivity analysis; therefore, its robustness should be considered not absolute. In general, different reviews and meta-analysis did not find any association between diabetes and hospital-acquired pneumonia^13,47^. Other studies reported that diabetes mellitus is a protective factor against acute respiratory distress syndrome (ARDS), probably due to a lowering effect on cytokine storm produced by diabetes in this context^48,49^. Furthermore, a recent pre-clinical study suggests a potential protective effect of metformin, the most used oral antidiabetic drug, against severe forms of pneumonia due to Streptococcus pneumoniae^50^. Taken together, the above-mentioned findings might furnish a possible interpretative key of the negative association between diabetes mellitus and the incidence of nosocomial pneumonia found in our study. Probably, in our cohort, diabetes mellitus has a protective effect only on moderate-severe forms of pneumonia, which were likely the most frequently diagnosed. Indeed, it is probable that mild forms of pneumonia were sometimes not diagnosed due to the suboptimal sensitivity of radiological investigations in our setting, because of the frequent use of bedside chest radiography, mainly for patients hospitalized at the Stroke Unit^51^. On this topic, also results taken from models including patients without overlapping diagnoses, are interesting (see supplementary materials). In these models, diabetes did not result a protective factor for healthcare acquired pneumonia. This finding suggests that diabetes might be protective only in patients with other predisposing conditions for healthcare-acquired pneumonia, first of all stroke, but probably it per se does not significantly modify the risk for this kind of nosocomial infection. In summary, based on our data, we can reasonably exclude that diabetes mellitus is a risk factor for nosocomial pneumonia in geriatric neurological settings, but we cannot state with certainty that it is a protective factor for pneumonia, regardless of its severity.

The univariate analysis showed that the use of antipsychotic drugs was a risk factor for nosocomial infections (globally considered) and for bloodstream infections; however, utilizing different models of multivariable analysis, this association was confirmed only for bloodstream infections. Sensitivity analysis found an OR higher than 1 for antipsychotic drugs in both people with and without dementia, with respect to bloodstream infections. However, only the association between bloodstream infections and these treatments in people without dementia resulted to be statistically significant. Interestingly, the links between antipsychotic drugs and bloodstream infections were previously reported by another study performed in Argentina, including a cohort of patients with a chronic obstructive pulmonary disease and a mean age of 75 years^52^. In literature a link between these medications and an increased risk of urinary tract infections was also reported: in our study, we only found a trend towards this association, but it did not reach statistical significance^53^. Analogously, we did not observe a clear relationship with pneumonia. However, the abundant representation in our cohort of major predisposing conditions may delete the effect of antipsychotic medications regarding both nosocomial urinary tract infections and healthcare-acquired pneumonia.

The putative link of antipsychotic drugs with bloodstream infections may be explained by the probable immunological dysfunction caused by antipsychotic medications, as showed by a recent pre-clinical study^54^. With respect to the result of sensitivity analysis on bloodstream infections, we invite the reader to consider the very low number of events among people with dementia (6 only!), as it may probably be sufficient to explain the failure to achieve the statistical significance. In general we think that, considering the low numbers of events, it is not possible to exclude that the association between antipsychotic medications and bloodstream infections is a false positive result; on the other hand, the fact that other studies have reported the same finding makes this relationship plausible.

Our study presents some limitations. First of all, the present is a retrospective study and due to its design, by definition it is affected by an intrinsic risk of bias^55^. However, in order to reduce the risk typically affecting retrospective studies, and in particular those based on electronic databases, i.e. that missing information could affect the final results, here the Authors have checked all medical records before performing statistical analysis. A second limitation is intrinsically related to the topic of the study, i.e. nosocomial infections among elderly patients with neurological diseases. To this regard, it is important to underline that is often difficult to distinguish between an asymptomatic bacteriuria and a urinary tract infection symptomatic for delirium in an elderly subject presenting multiple conditions potentially able to cause delirium (medications, neurological diseases, etc.). Not even the presence of fever is sufficient to render symptomatic a bacteriuria, because in a geriatric neurological setting many conditions can cause fever, among which we should not forget the fever of central origin. The lack of a universally accepted definition of nosocomial infections, with particular regard to nosocomial urinary tract infections, could affect the reproducibility of the present results. Another aspect potentially limiting the generalizability of our results is the fact that the present is a monocentric study performed at a tertiary neurologic Clinic specialized in the management of geriatric patients with neurological diseases: this indeed could be a source of selection bias. Finally, regarding blood infections, the Event per Variable (EPV) ratio is small, leading to a potential risk of false positive and false negative findings: in any case the Firth’s penalized model adopted in the present study was proven as one of the best solution to reduce biases^56,57^.

All the above considered, our study has also some strengths. First, the sample study was relatively large (1543 patients). Second, we applied different models of multivariable analysis in order to reduce, as far as possible, the impact of confounding factors. Third, we performed a sensitivity analysis to check the robustness of the results.

In conclusion, our study suggests that, among geriatric patients with neurological diseases hospitalized at a Tertiary Hospital, stroke, age, dementia the use of medical devices (urinary catheter, vascular catheters and nasogastric tube) and antipsychotic medications represent risk factors for nosocomial infections. The practical implications of these findings are quite obvious: in order to reduce the risk of nosocomial infections, clinicians should reduce, as far as possible, the use of medical devices and antipsychotic medications in a geriatric neurological setting. Further prospective multicentric studies should be performed to verify these results.

### Supplementary Information

Supplementary Table S1.

## Data Availability

The datasets used and/or analysed during the current study are available from the corresponding author on reasonable request.
