# Supplementary material for: Dementia, stroke, age, use of medical devices and antipsychotic drugs may increase the risk of nosocomial infections among elderly patients hospitalized at Neurology Clinics
Source: Sci Rep. 2023 Oct 31;13:18687. doi: 10.1038/s41598-023-46102-2 (PMC10618180; doi:10.1038/s41598-023-46102-2)
Supplement: Supplementary file 1 — Supplementary Table S1. [file 41598_2023_46102_MOESM1_ESM.docx]

**Table S1: Risk and protective factors for nosocomial infections for principal diagnoses: all, pneumonia, urinary-tract infection and bloodstream infection (N=1284)**

| **MODEL 1** | | | **MODEL 2** | | | **MODEL 3** | | |
| --- | --- | --- | --- | --- | --- | --- | --- | --- |
| **OUTCOME: NOSOCOMIAL INFECTIONS** |  |  |  |  |  |  |  |  |
|  | **OR(95%CI)** | **p** |  | **OR(95%CI)** | **p** |  | **OR(95%CI)** | **p** |
| Sex M | 1.24(0.91-1.67) | 0.169 | Sex M | 1.38(1.00-1.91) | 0.051 | Sex M | 1.24(0.90-1.71) | 0.187 |
| Age | 1.08(1.05-1.10) | **<0.001** | Age | 1.06(1.03-1.09) | **<0.001** | Age | 1.07(1.05-1.10) | **<0.001** |
| Dementia | 1.24(0.87-1.76) | 0.233 | Dementia | 1.04(0.70-1.53) | 0.857 | Dementia | 1.11(0.76-1.64) | 0.584 |
| Stroke | 1.91(1.23-2.95) | **0.004** | Stroke | 1.84(1.15-2.95) | **0.012** | Stroke | 1.97(1.24-3.13) | **0.004** |
| Diabetes | 1.25(0.76-2.06) | 0.371 | Diabetes | 1.33(0.79-2.25) | 0.282 | Diabetes | 1.30(0.77-2.19) | 0.322 |
|  |  |  | Antipsychotic drugs | 1.00(0.64-1.57) | 0.993 | Antipsychotic drugs | 1.02(0.65-1.61) | 0.925 |
|  |  |  | CVC | 3.35(1.23-9.11) | **0.018** | days CVC | 1.01(0.97-1.05) | 0.623 |
|  |  |  | Power Glide | 0.38(0.05-2.63) | 0.326 | days Power Glide | 1.05(0.62-1.79) | 0.849 |
|  |  |  | PVC | 1.62(1.14-2.30) | **0.007** | days PVC | 1.00(1.00-1.00) | 0.710 |
|  |  |  | PICC | 0.86(0.33-2.26) | 0.766 | days PICC | 1.00(0.98-1.02) | 0.885 |
|  |  |  | Nasogastric tube | 3.23(1.58-6.60) | **0.001** | Days Nasogastric tube | 1.02(0.98-1.06) | 0.309 |
|  |  |  | Midline | 3.06(1.37-6.82) | **0.006** | days Midline | 1.14(0.98-1.32) | 0.093 |
|  |  |  | Urinary Catheter | 5.11(3.34-7.81) | **<0.001** | Days Urinary Catheter | 1.08(1.06-1.09) | **<0.001** |
| _cons | 0.00(0.00-0.00) | <0.001 | _cons | 0.00(0.00-0.00) | <0.001 | _cons | 0.00(0.00-0.00) | <0.001 |
| **OUTCOME: PNEUMONIA** |  |  |  |  |  |  |  |  |
|  | **OR(95%CI)** | **p** |  | **OR(95%CI)** | **p** |  | **OR(95%CI)** | **p** |
| Sex M | 2.24(1.53-3.29) | **<0.001** | Sex M | 2.55(1.71-3.80) | **<0.001** | Sex M | 2.28(1.54-3.36) | **<0.001** |
| Age | 1.07(1.04-1.10) | **<0.001** | Age | 1.06(1.03-1.10) | **<0.001** | Age | 1.07(1.04-1.10) | **<0.001** |
| Dementia | 1.38(0.89-2.14) | 0.155 | Dementia | 1.27(0.80-2.02) | 0.312 | Dementia | 1.27(0.80-2.02) | 0.308 |
| Stroke | 1.84(1.07-3.16) | **0.026** | Stroke | 1.71(0.99-2.96) | 0.056 | Stroke | 1.73(1.00-2.98) | **0.048** |
| Diabetes | 0.83(0.41-1.68) | 0.602 | Diabetes | 0.84(0.41-1.72) | 0.637 | Diabetes | 0.82(0.40-1.66) | 0.580 |
|  |  |  | Antipsychotic drugs | 1.00(0.58-1.72) | 0.999 | Antipsychotic drugs | 0.98(0.57-1.68) | 0.937 |
|  |  |  | Nasogastric tube | 7.74(3.95-15.16) | **<0.001** | days Nasogastric tube | 1.07(1.04-1.10) | **<0.001** |
| _cons | 0.00(0.00-0.00) | <0.001 | _cons | 0.00(0.00-0.00) | <0.001 | _cons | 0.00(0.00-0.00) | <0.001 |

**Abbreviations:** CVC = central intravenous catheter, PVC = peripheral intravenous catheter, PICC= peripherally inserted central catheter.

**Table S1 (cont.)**

| **OUTCOME: URINARY-TRACT INFECTION** |  |  |  |  |  |  |  |  |
| --- | --- | --- | --- | --- | --- | --- | --- | --- |
|  | **OR(95%CI)** | **p** |  | **OR(95%CI)** | **p** |  | **OR(95%CI)** | **p** |
| Sex M | 0.63(0.42-0.95) | **0.027** | Sex M | 0.64(0.42-0.97) | **0.035** | Sex M | 0.61(0.40-0.93) | **0.021** |
| Age | 1.07(1.04-1.10) | **<0.001** | Age | 1.06(1.03-1.09) | **<0.001** | Age | 1.07(1.04-1.10) | **<0.001** |
| Dementia | 0.89(0.56-1.40) | 0.609 | Dementia | 0.81(0.50-1.31) | 0.383 | Dementia | 0.80(0.49-1.30) | 0.364 |
| Stroke | 1.66(0.98-2.82) | 0.060 | Stroke | 1.80(1.04-3.12) | **0.036** | Stroke | 1.69(0.97-2.92) | 0.062 |
| Diabetes | 1.24(0.68-2.28) | 0.486 | Diabetes | 1.31(0.70-2.47) | 0.401 | Diabetes | 1.36(0.73-2.53) | 0.338 |
|  |  |  | Antipsychotic drugs | 1.27(0.75-2.17) | 0.377 | Antipsychotic drugs | 1.27(0.74-2.16) | 0.383 |
|  |  |  | Urinary Catheter | 5.89(3.81-9.12) | **<0.001** | Days Urinary Catheter | 1.06(1.05-1.08) | **<0.001** |
| _cons | 0.00(0.00-0.01) | <0.001 | _cons | 0.00(0.00-0.02) | <0.001 | _cons | 0.00(0.00-0.01) | <0.001 |
| **OUTCOME: BLOODSTREAM INFECTION** |  |  |  |  |  |  |  |  |
|  | **OR(95%CI)** | **p** |  | **OR(95%CI)** | **p** |  | **OR(95%CI)** | **p** |
| Sex M | 0.66(0.22-2.02) | 0.468 | Sex M | 0.70(0.22-2.20) | 0.544 | Sex M | 0.91(0.28-2.91) | 0.868 |
| Age | 1.03(0.95-1.11) | 0.520 | Age | 1.03(0.95-1.12) | 0.489 | Age | 1.01(0.93-1.11) | 0.783 |
| Dementia | 0.87(0.26-2.94) | 0.828 | Dementia | 0.55(0.15-2.03) | 0.368 | Dementia | 0.43(0.11-1.74) | 0.240 |
| Stroke | 2.31(0.67-8.00) | 0.186 | Stroke | 2.24(0.61-8.13) | 0.222 | Stroke | 1.54(0.38-6.26) | 0.546 |
| Diabetes | 0.28(0.02-5.07) | 0.392 | Diabetes | 0.30(0.02-5.47) | 0.420 | Diabetes | 0.00(0.00-2.25) | 0.072 |
|  |  |  | Antipsychotic drugs | 3.93(1.23-12.57) | **0.021** | Antipsychotic drugs | 4.26(1.22-14.90) | **0.023** |
|  |  |  | CVC | 10.52(2.26-49.04) | **0.003** | days CVC | 1.19(1.05-1.35) | **0.007** |
|  |  |  | Power Glide | 8.18(0.36-188.47) | 0.189 | days Power Glide | 1.83(1.11-3.03) | **0.018** |
|  |  |  | PVC | 0.86(0.28-2.66) | 0.797 | days PVC | 1.00(1.00-1.00) | **0.045** |
|  |  |  | PICC | 1.15(0.06-21.32) | 0.925 | days PICC | 1.02(1.00-1.04) | 0.093 |
|  |  |  | Midline | 9.72(2.63-35.95) | **0.001** | days Midline | 1.26(1.08-1.47) | **0.004** |
| _cons | 0.00(0.00-2.93) | 0.097 | _cons | 0.00(0.00-2.37) | 0.082 | _cons | 0.00(0.00-8.46) | 0.157 |

**Abbreviations:** CVC = central intravenous catheter, PVC = peripheral intravenous catheter, PICC= peripherally inserted central catheter.
